# DrugShot: querying biomedical search terms to retrieve prioritized lists of small molecules

**DOI:** 10.1186/s12859-022-04590-5

**Published:** 2022-02-19

**Authors:** Eryk Kropiwnicki, Alexander Lachmann, Daniel J. B. Clarke, Zhuorui Xie, Kathleen M. Jagodnik, Avi Ma’ayan

**Affiliations:** Department of Pharmacological Sciences, Icahn School of Medicine at Mount Sinai, One Gustave L. Levy Place, Box 1603, New York, NY 10029 USA

**Keywords:** Drug repurposing, Text mining, Machine learning, Search engine, Transcriptomics

## Abstract

**Background:**

PubMed contains millions of abstracts that co-mention terms that describe drugs with other biomedical terms such as genes or diseases. Unique opportunities exist for leveraging these co-mentions by integrating them with other drug-drug similarity resources such as the Library of Integrated Network-based Cellular Signatures (LINCS) L1000 signatures to develop novel hypotheses.

**Results:**

DrugShot is a web-based server application and an Appyter that enables users to enter any biomedical search term into a simple input form to receive ranked lists of drugs and other small molecules based on their relevance to the search term. To produce ranked lists of small molecules, DrugShot cross-references returned PubMed identifiers (PMIDs) with DrugRIF or AutoRIF, which are curated resources of drug-PMID associations, to produce an associated small molecule list where each small molecule is ranked according to total co-mentions with the search term from shared PubMed IDs. Additionally, using two types of drug-drug similarity matrices, lists of small molecules are predicted to be associated with the search term. Such predictions are based on literature co-mentions and signature similarity from LINCS L1000 drug-induced gene expression profiles.

**Conclusions:**

DrugShot prioritizes drugs and small molecules associated with biomedical search terms. In addition to listing known associations, DrugShot predicts additional drugs and small molecules related to any search term. Hence, DrugShot can be used to prioritize drugs and preclinical compounds for drug repurposing and suggest indications and adverse events for preclinical compounds. DrugShot is freely and openly available at: https://maayanlab.cloud/drugshot and https://appyters.maayanlab.cloud/#/DrugShot.

**Supplementary Information:**

The online version contains supplementary material available at 10.1186/s12859-022-04590-5.

## Background

The amount of published biomedical literature available online is expanding at an increasing rate [1, 2]. With this growing corpus of knowledge, unique opportunities exist for synthesizing the available information using text-mining and leveraging these synthesized data to develop novel hypotheses. Previous efforts have created text-mining search engines for querying the PubMed database to retrieve ranked lists of biological and chemical entities associated with a search term of interest based on co-mentions [3–7]. Such methods make it more convenient for researchers to explore the vast literature space by returning an aggregated knowledge report from specific subdomains of research. For example, Finding Associated Concepts with Text Analysis (FACTA+) enables users to query arbitrary search terms to retrieve biomedical concepts such as genes, diseases, symptoms, drugs, enzymes, and compounds associated with the search term based on shared PMIDs [3]. For each of the returned biomedical concepts, a report of specific journal articles detailing the association between the query term and the biomedical concept is provided. Similarly, KinderMiner Web [4] ranks the associations among a query phrase and a list of target terms using an index derived from PubMed. The KinderMiner algorithm employs string matching and co-occurrence counting. DigSee [5] associates genes, diseases, and biological events by indexing PubMed abstracts and using graph-based feature sets. This platform returns a ranked list of 'evidence sentences' that describe the genes involved in a disease. Biomedical Entity Search Tool (BEST) [6] employs a dictionary-based indexing strategy that covers 12 databases including PubMed. For each query, BEST returns 10 categories of associated ranked biomedical entities including genes, drugs, chemical compounds, targets, and diseases. PolySearch2 [7] supports queries of the form 'Given X, report all associated Ys', where X and Y are biological entities that can include genes, proteins, drugs, and drug actions. The PolySearch2 platform surveys a range of platforms including PubMed, and 14 additional biomedical databases, as well as Wikipedia and US patent application abstracts, and returns ranked lists of relevant results.

The ability to retrieve relevant biological entities from the results of a literature query opens opportunities for discovery. By abstracting the relationships among biological entities, and incorporating other data resources, it is possible to create knowledge graphs [8–10]. Pathway Assembly from Literature Mining - an Information Search Tool (PALM-IST) is a search engine that conducts text searches to retrieve relevant biological entities from literature but goes a step further to assemble protein and pathway interaction networks based on the extracted entities from the text [8]. LitPathExplorer [9] queries PubMed for user-specified biological entities, roles, and events, and uses semi-supervised learning to discover new associations and events. The LitPathExplorer platform provides interactive visualizations that permit the exploration of pathway models. LitPathExplorer content is organized based on levels of confidence in the reported relationships. Geneshot [10], a tool that we developed, enables users to query biomedical terms to retrieve top-ranked genes based on shared PMIDs. Additionally, Geneshot further prioritizes genes associated with the search term based on co-occurrence and co-expression with the top returned genes from the literature search. Leveraging the literature-associated genes to make further predictions permits the creation of meta-networks that may uncover unknown gene-function associations. Drug discovery is a field that may benefit from such approaches to prioritize novel compounds for treatment of diseases, or for the discovery of previously unknown associations between small molecules and side effects, biological pathways, and other attributes.

Here we present DrugShot, a web-based search engine and an Appyter [11] that enables users to query any biomedical or biological term to retrieve sets of small molecules associated with the search term. Upon submitting a query, DrugShot generates a variety of figures and tables that display the most relevant compounds to the queried search term. DrugShot predicts additional compounds based on literature co-mentions of small molecules within PubMed abstracts, and signature similarity based on LINCS L1000 drug-induced gene expression signatures [12]. By utilizing available biomedical literature together with drug-drug association resources, DrugShot presents unique opportunities for discovering new annotations for drugs and preclinical small molecules.

## Implementation

### Creating DrugRIF and AutoRIF

A master list of experimental and approved small molecule names and corresponding International Union of Pure and Applied Chemistry (IUPAC) Chemical Identifier Keys (InChIKeys) [13] was curated from Drugmonizome [14] and SEP-L1000 [15]. Amino acids, chemical reagents, metabolites, and other non-drug-like molecules were manually pruned from the master list of compounds as these entities are commonly overrepresented in the literature. To create DrugRIF, one of the two underlying drug-PMID association databases used by DrugShot, a total of 1,996,791 drug-publication associations covering 2,346 approved drugs and 3,845 preclinical small molecules were extracted from PubChem. Every small molecule in the master list was queried by the InChIKey through the cross-referencing endpoint from the PubChem PUG-REST application programming interface (API) [16, 17] to retrieve PMIDs associated with each small molecule. To create AutoRIF, an alternate database that contains a total of 7,977,179 drug-publication associations covering 2,627 approved drugs and 3,732 preclinical small molecules, each small molecule name from the master list was harmonized with a Medical Subject Heading (MeSH) preferred term identifier. These terms were queried using the National Center for Biotechnology Information (NCBI) E-utilities API to query PubMed with the term [18]. The search returned PubMed IDs associated with each MeSH small molecule term. It should be noted that we filtered the results to retrieve only publications that include the searched MeSH term. Small molecule terms were required to be associated with at least two PMIDs to be included in DrugRIF or AutoRIF. DrugRIF is a manually curated resource of drug-PMID relationships where the provenance of each entity can be traced to a PubChem record, whereas AutoRIF is a more comprehensive resource, albeit more prone to errors because it relies on fuzzier string matching to retrieve PubMed-ID/drug associations.

### Creating the prediction matrices

Two separate DrugRIF and AutoRIF literature co-mentions prediction matrices were created, one from the DrugRIF file and one from the AutoRIF file. These two separate matrices were created by taking pairwise absolute counts of shared PMIDs between the 6,151 (for DrugRIF) and 6,360 (for AutoRIF) drugs and small molecules. Drug-drug similarity was also computed from pre-processed LINCS L1000 drug-induced gene expression signatures stored in the SEP-L1000 database [15]. Pairwise cosine similarity was used to compute similarity between all drug-induced signatures, producing a similarity matrix containing 19,679 drugs and preclinical small molecules.

### The DrugShot search engine

**Fig. 1:**
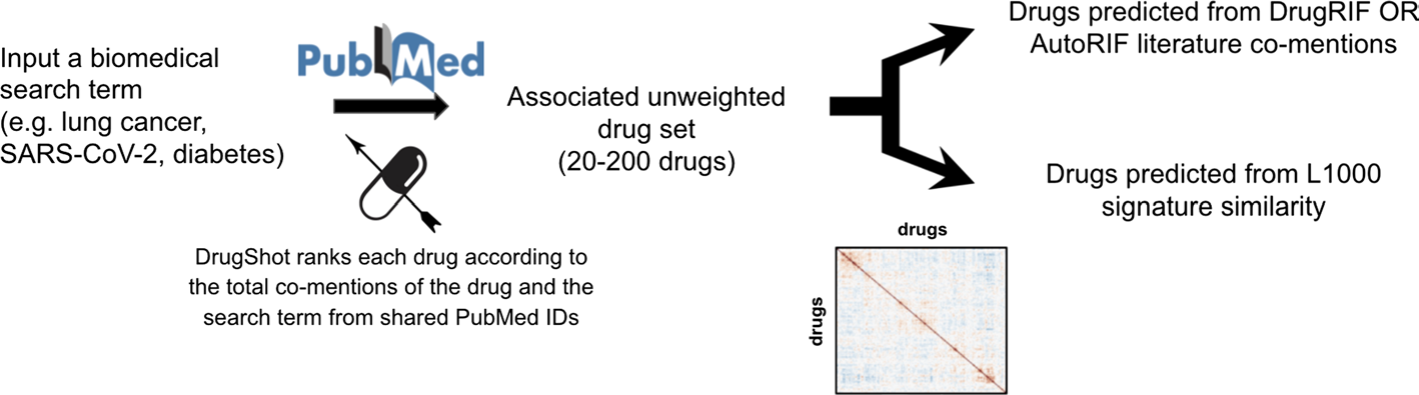
A graphical schema of the DrugShot workflow

The DrugShot search engine enables users to input any search term. The term is queried using the NCBI E-utilities E-search PubMed API [18] to retrieve PMIDs associated with the search term. The term-PMID associations are then compared with the drug-PMID associations in DrugRIF or AutoRIF to produce a ranked list of small molecules that share PMIDs with the search term. The search results table includes counts of the total number of overlapping PMIDs between the small molecule and the search term in addition to the normalized fraction of publications mentioning the small molecule and search term divided by the number of publications that mention the small molecule regardless of the search term. An unweighted drug set is created from the ranked small molecules from the returned list by a specified cutoff. The small molecules included in the set are ranked by the product of the total associated publications multiplied by their normalized fraction. This approach considers how frequently a small molecule is co-mentioned with the search term in the literature, as well as how specific the term is to a particular small molecule. The literature co-mentions and signature similarity prediction matrices are seeded with the unweighted drug set, and the top predicted compounds are ranked by their average similarity to the small molecules in the unweighted drug set (Fig. 1). As an additional option, users may also submit their own unweighted drug sets using the DrugShot augmentation option.

## Results

### Interacting with the DrugShot web-based application

**Fig. 2:**
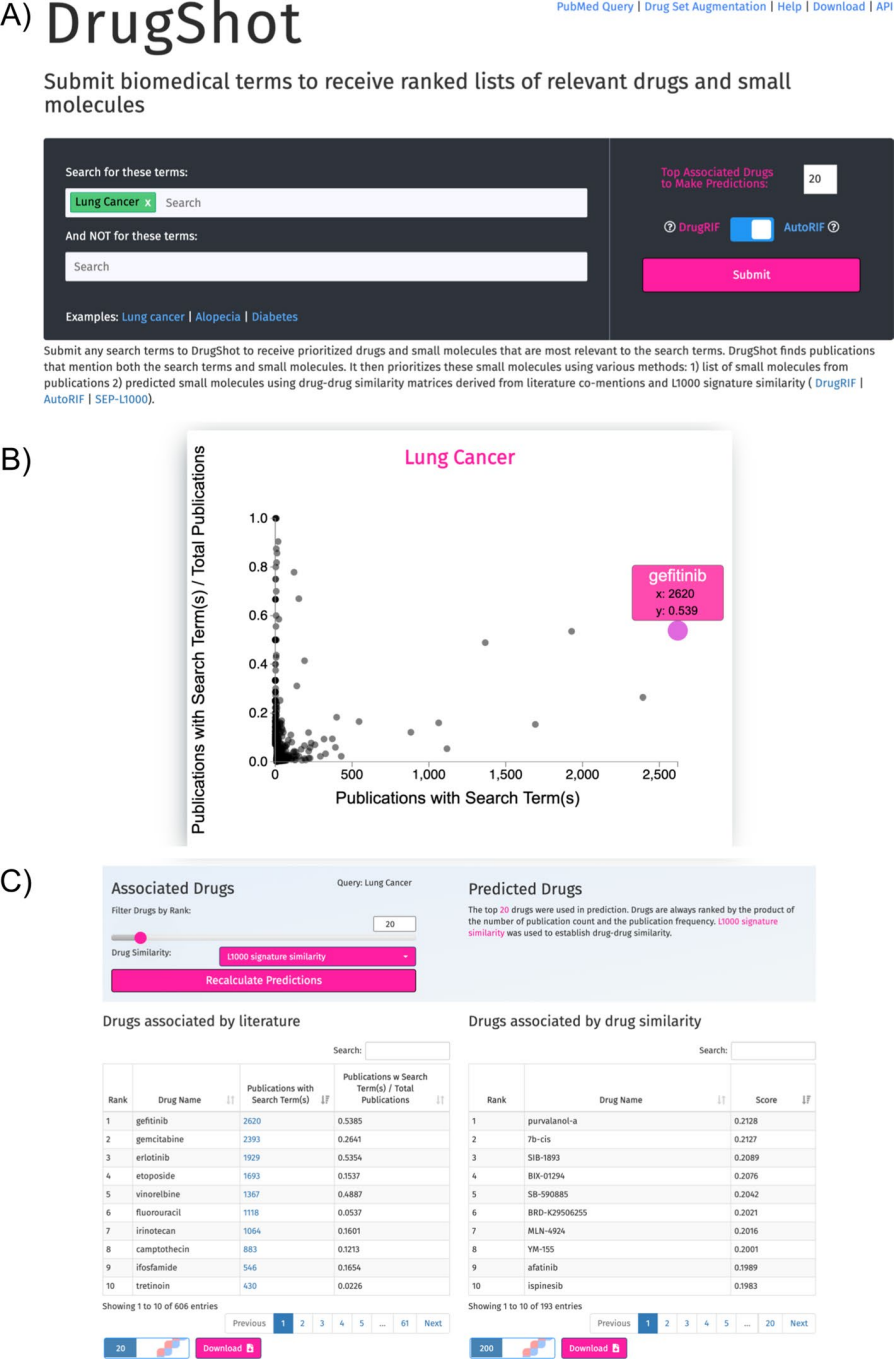
DrugShot web application user interface. **A** Input form section for querying a biomedical search term of interest. **B** Scatter plot of all publications that mention both the drug and the search terms against the normalized values. **C** Tables providing a ranked list of associated drugs from DrugRIF (left), and predictions based on signature similarity (right)

DrugShot is available as a web-based application with a user-friendly interface. The DrugShot landing page is divided into three sections (Fig. 2). The first section contains the input form for submitting Search terms (Fig. 2A). The top text box is for submitting search terms that can be combined with a logical AND operator, while the bottom text box is for terms that should not be included in the search. Once the user presses the search button, publications are retrieved from PubMed, and if excluding terms are entered, these are filtered based on the exclusion criteria. The returned PMIDs are then cross-referenced against DrugRIF or AutoRIF. The second section contains an interactive scatter plot that visualizes the returned search results (Fig. 2B). The scatter plot displays the total matching publications for each small molecule on the x-axis, and a normalized fraction of matching publications that mention the small molecule with the search terms divided by the total publications that mention the small molecule regardless of the search terms on the y-axis. Hovering over any point in the scatter plot displays more detailed information about each small molecule. The bottom section of the DrugShot landing page displays the returned results in interactive downloadable tables (Fig. 2C). On the left is a table that contains a ranked list of small molecules associated with the search term, while on the right is another table with ranked small molecules that are predicted to be related to the search term based on literature co-mentions or gene expression signature similarity. Small molecules from both tables can be submitted to DrugEnrichr (https://maayanlab.cloud/DrugEnrichr/) for drug set enrichment analysis, and the full content of both tables is available for download as tab-separated values (TSV) files. The DrugShot website also includes a drug set augmentation feature where users can submit their own unweighted set of small molecules to be augmented with additional compounds based on literature co-mentions or LINCS L1000 gene expression signature similarity.

### Interacting with the DrugShot Appyter

**Fig. 3:**
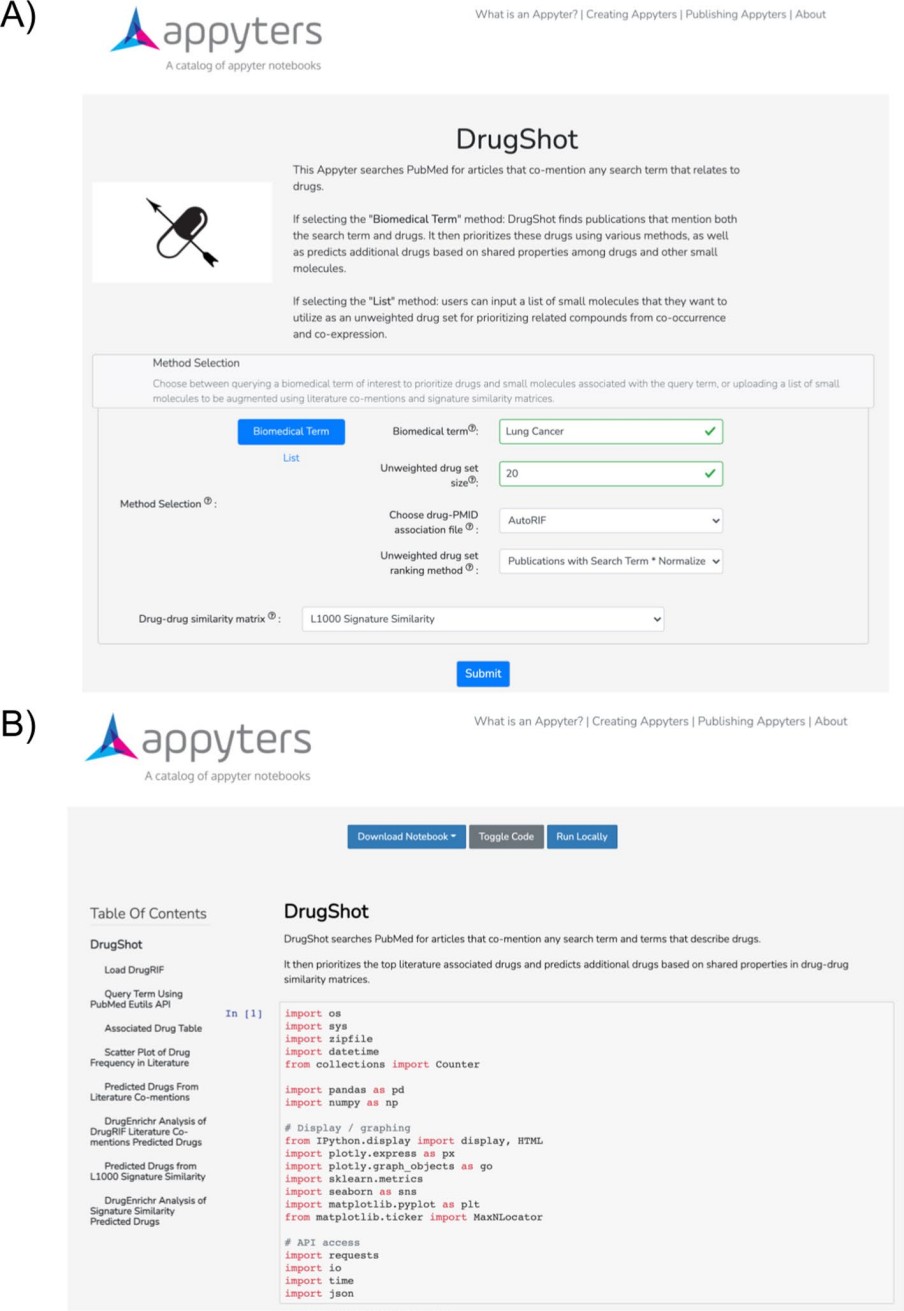
The DrugShot Appyter. **A** Input form where the user can select a biomedical term of interest, unweighted drug set size, the database of drug-PMID associations, and the method to rank the small molecules from the unweighted drug set. Additionally, the user can select which drug-drug similarity matrix to use to make predictions. **B** The executed notebook with options for download, toggling code, and running the notebook locally. Each of the elements in the table of contents is interactive for easy navigation of the Appyter notebook.

The DrugShot Appyter is an alternative implementation of DrugShot. The Appyter first presents a simple input form that requires the user to enter a search term and an integer ranging from 20-200 (Fig. 3A). This integer corresponds to the size of the unweighted drug set that will be used for making predictions about additional compounds that might be relevant to the search term. Furthermore, there are drop-down menus to select the drug-PMID association file, the method to rank the small molecules included in the unweighted drug set, and lastly, the drug-drug similarity matrix to use for making predictions. Alternatively, users may upload their own set of small molecules for drug set augmentation with the literature co-mentions and gene expression signature similarity matrices. Once the input form of the Appyter is filled and the user presses submit, the Appyter with the user-specified inputs is executed in the cloud (Fig. 3B). The user is first presented with a downloadable table that displays the top 20 small molecules associated with the search term based on the number of shared PMIDs based on DrugRIF or AutoRIF, as well as a scatter plot of drug frequencies in the literature. The unweighted drug set is created from the top-ranked associated small molecules, and it is used for the subsequent predictions using either the literature co-mentions or LINCS L1000 gene expression signature similarity matrices. Both the literature co-mentions and the signature similarity results are provided in different sections of the Appyter report. Additionally, downloadable tables of the top 20 predicted compounds from the literature co-mentions and the signature similarity are produced in the respective sections of the Appyter report. Lastly, the DrugShot Appyter reports selected drug set enrichment results generated by DrugEnrichr as bar charts that display the top enriched terms for the submitted drug sets. Permanent links to the full DrugEnrichr drug set enrichment analysis results are also made available.

### Benchmarking DrugShot predictions

**Fig. 4:**
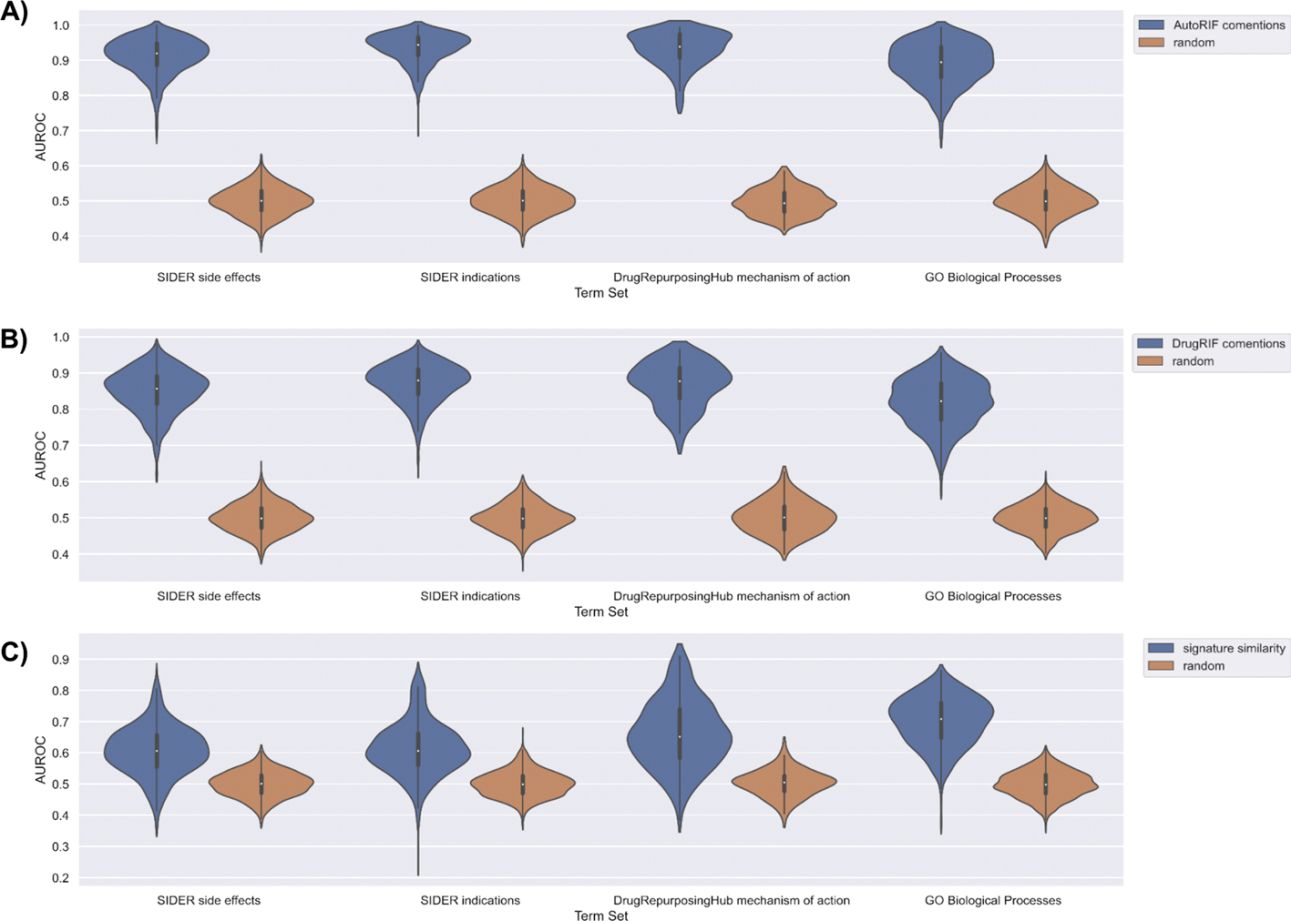
Violin plots of AUROC distributions for each collection of search terms for each prediction matrix compared with random shuffles of the prediction matrix. AUROCs for each term were determined based on the rankings of the unweighted drug set created from AutoRIF in each prediction matrix. **A** AutoRIF literature co-mentions prediction matrix. **B** DrugRIF literature co-mentions prediction matrix. **C** Signature similarity prediction matrix

**Fig. 5:**
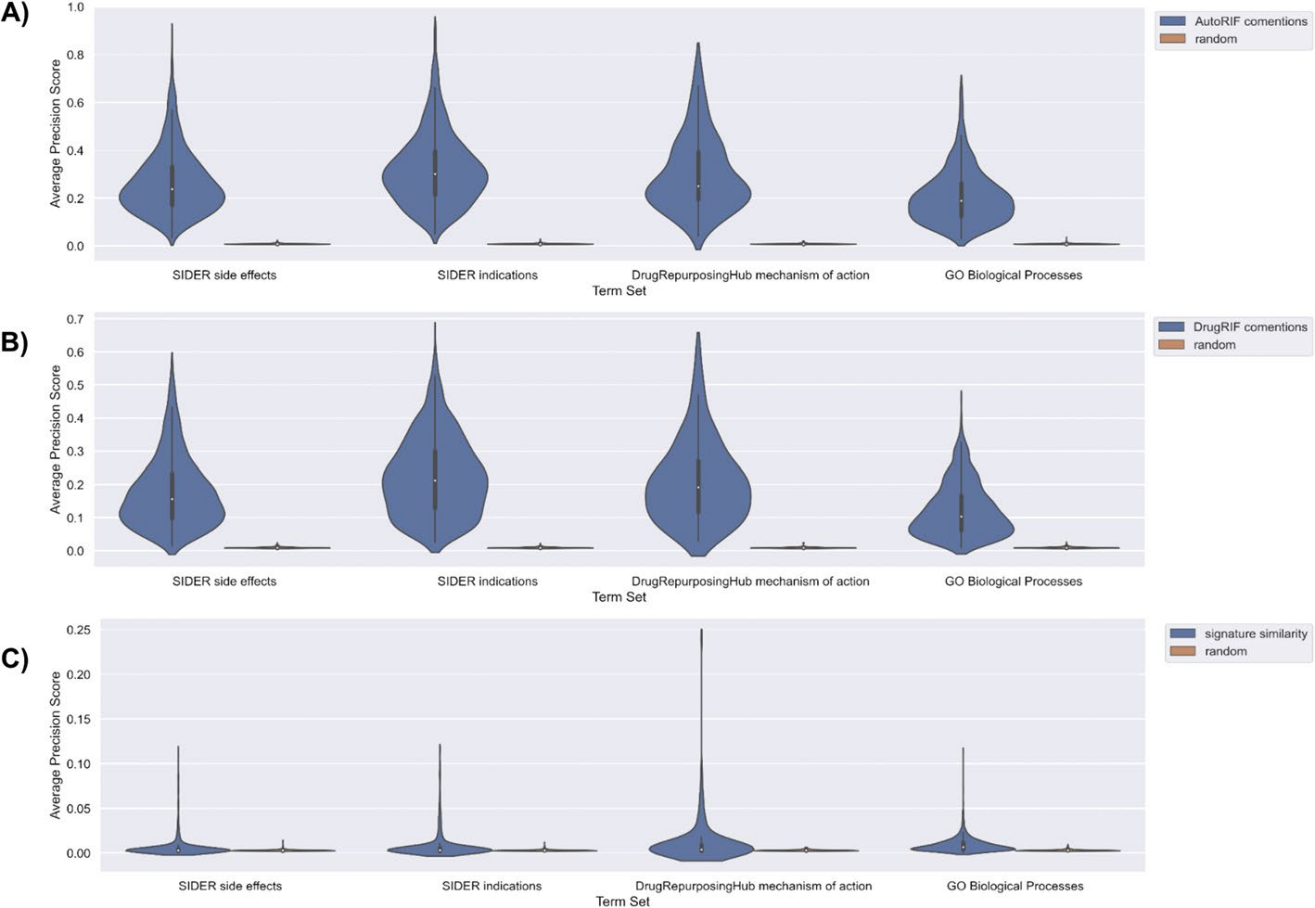
Violin plots of average precision score distributions for each collection of search terms for each prediction matrix compared with random shuffles of the prediction matrix. Average precision scores for each term were determined based on the rankings of the unweighted drug set created from AutoRIF in each prediction matrix. **A** AutoRIF literature co-mentions prediction matrix. **B** DrugRIF literature co-mentions prediction matrix. **C** Signature similarity prediction matrix

**Fig. 6:**
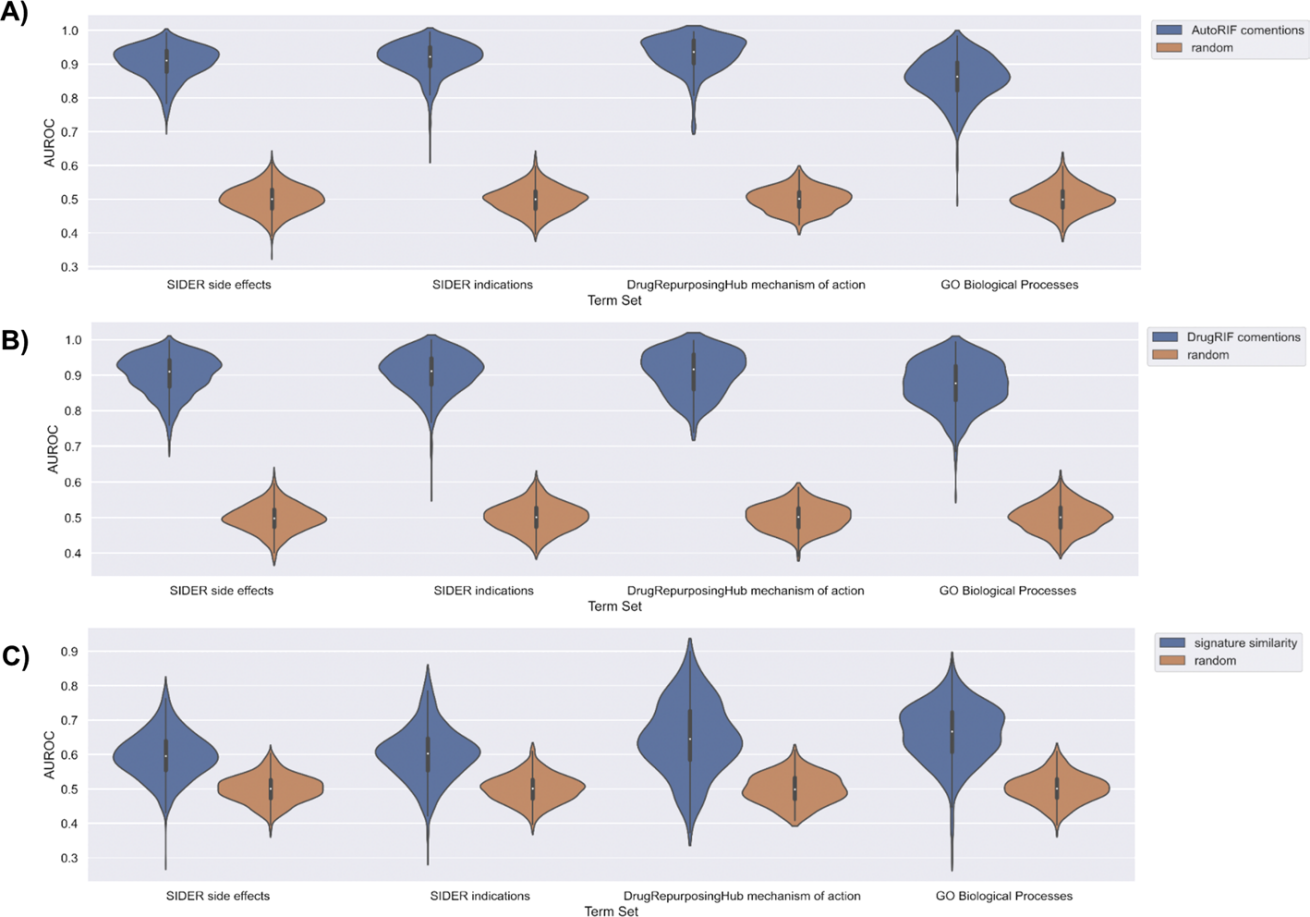
Violin plots of AUROC distributions for each collection of search terms for each prediction matrix compared with random shuffles of the prediction matrix. AUROCs for each term were determined based on the rankings of the unweighted drug set created from DrugRIF in each prediction matrix. **A** AutoRIF literature co-mentions prediction matrix. **B** DrugRIF literature co-mentions prediction matrix. **C** Signature similarity prediction matrix

**Fig. 7:**
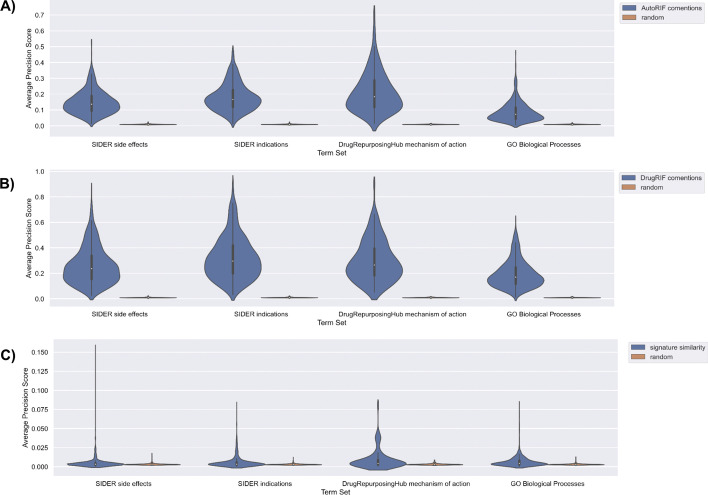
Violin plots of average precision score distributions for each collection of search terms for each prediction matrix compared with random shuffles of the prediction matrix. Average precision scores for each term were determined based on the rankings of the unweighted drug set created from DrugRIF in each prediction matrix. **A** AutoRIF literature co-mentions prediction matrix. **B** DrugRIF literature co-mentions prediction matrix. **C** Signature similarity prediction matrix

To benchmark the predictions made by DrugShot, we queried DrugShot with 835 drug indication terms and 1,298 side effect terms from SIDER [19], 154 mechanism of action terms from Drug Repurposing Hub [20], and 1,364 Gene Ontology Biological Processes terms [21]. We then examined how well the predictions made by DrugShot recover known associations between drugs and terms. To benchmark the predictions made by the literature co-mentions and the LINCS L1000 gene expression signature similarity matrices, an unweighted drug set from AutoRIF or DrugRIF was created for each queried term. We then calculated the average area under the receiver operating characteristic curve (AUROC) and the average precision-recall curves (PRC) to assess the ability of each prediction matrix, literature co-mentions, and drug similarity based on gene expression, to rank the small molecules from the unweighted drug set (Figs. 4, 5, 6 and 7). To verify that the signature similarity methods and matrices perform better than random, we computed the Mann-Whitney U-statistic for the AUROCs and PRCs generated from each library of drug-related terms compared with what is expected for a random classifier (Tables 1 and 2). While the predictions based on literature co-mentions appear to perform much better than the predictions made by drug-drug similarity based on drug-induced gene expression signatures, the predictions based on gene expression signatures should be considered more novel. In addition, while in general the predictions based on co-expression produced AUROCs that are below 0.7 and PRCs that are indistinguishable from those produced for random classifiers, many predictions produced much higher AUROCs and PRCs.

**Table 1:** Mann-Whitney U statistic and *p*-values calculated from Mann-Whitney U test to determine a significant difference in average AUROCs and PRCs of signature similarity matrix rankings of the unweighted drug set created from AutoRIF and randomly shuffled predictions across libraries of drug-related terms that were queried using DrugShot

Library	AUROC evaluation		PRC evaluation	
	U-statistic	*p*-value	U-statistic	*p*-value
SIDER side effects (1298 terms)	1,495,996	9.12E−257	922,468	2.75E−05
SIDER indications (835 terms)	626,342	8.62E−175	402,517	4.48E−08
Drug repurposing hub MoA (154 terms)	20,213	2.82E−36	14,484	1.62E−06
GO biological processes (1364 terms)	898,342	6.53E−295	776,522	7.79E−156

**Table 2:** Mann Whitney U statistic and p-values calculated from Mann-Whitney U test to determine a significant difference in average AUROCs and PRCs of signature similarity matrix rankings of the unweighted drug set created from DrugRIF and randomly shuffled predictions across libraries of drug-related terms that were queried using DrugShot

	AUROC evaluation		PRC Evaluation	
Library	U-statistic	*p*-value	U-statistic	*p*-value
SIDER side effects (1298 terms)	1,191,630	6.79E−240	771,508	1.37E−11
SIDER indications (835 terms)	558,470	1.39E−154	388,449	2.51E−15
Drug repurposing hub MoA (154 terms)	18,728	2.75E−32	14,061	1.74E−07
GO biological processes (1364 terms)	674,733	5.06E−230	505,036	6.32E−53

To further explore the predictions that produced the best AUROCs and PRCs using the LINCS L1000 gene expression similarity approach, we created DrugShot Appyter instances for the top 5 terms with the highest AUROCs from each collection of search terms (Table 3). The top performing terms are predominantly related to cancer, which is expected given that the small molecules were profiled in cancer cell lines. However, predictions for terms not specific to cancer are also predicted, for example, the side effects pancytopenia and gastrointestinal toxicity. It should be noted that there are many additional terms with high significance. The URLs in Table 3 provide persistent links to DrugShot Appyter instances each containing a detailed report about the respective query term in the table. Such predictions provide a rich foundation for hypotheses that could be further validated experimentally.

**Table 3:** Terms with highest performing AUROC values from the signature similarity matrix benchmark, each with a link to an Appyter instance

Term	AUROC	Library	DrugShot Appyter instance URLs
mTOR inhibitor	0.9042	Drug Repurposing Hub mechanism of action	https://appyters.maayanlab.cloud/DrugShot/9ba88a051c155dc0adcea183c52aebda3265e909/
AKT inhibitor	0.8953	Drug Repurposing Hub mechanism of action	https://appyters.maayanlab.cloud/DrugShot/d17a38351796e759557014ac14a6ef0098404d71/
Thymidylate synthase inhibitor	0.8821	Drug Repurposing Hub mechanism of action	https://appyters.maayanlab.cloud/DrugShot/4bac8288bbb11786b6cedb7aaae685e7a7957236/
PI3K inhibitor	0.8806	Drug Repurposing Hub mechanism of action	https://appyters.maayanlab.cloud/DrugShot/afab724e5461e367bd061684d4d1ecc7b96e4c13/
HDAC inhibitor	0.8785	Drug Repurposing Hub mechanism of action	https://appyters.maayanlab.cloud/DrugShot/61ebfcc11fe370a574041d9dfea7ab1f2d8a191e/
Small cell lung cancer	0.8683	SIDER indications	https://appyters.maayanlab.cloud/DrugShot/84d1f4fb47842d045d16c88801e421dfffee08f7/
Mitotic cytokinesis	0.8631	Gene Ontology	https://appyters.maayanlab.cloud/DrugShot/5ebe7c287b81769177694bfac65b3f6e72483961/
Doxorubicin metabolic process	0.8622	Gene Ontology	https://appyters.maayanlab.cloud/DrugShot/98e8263b587a47383e9d05381c0ebb4a67af78be/
Positive regulation of lymphoc. proliferation	0.8567	Gene Ontology	https://appyters.maayanlab.cloud/DrugShot/240cdbca4a8e45b83e55ffcdb1c1a2617c5da908/
Activation of MAPKKK activity	0.8554	Gene Ontology	https://appyters.maayanlab.cloud/DrugShot/295c6c5654acb00f8dcd493a0b5143215b3374ec/
Positive regulation of cell cycle arrest	0.8534	Gene Ontology	https://appyters.maayanlab.cloud/DrugShot/63483f95a344da35eb501b8b4da5b9602e88ad83/
Malignant glioma	0.84823	SIDER indications	https://appyters.maayanlab.cloud/DrugShot/98790408cbcbaa0cc86d969cff3871e699c115ef/
Pancytopenia	0.8448	SIDER indications	https://appyters.maayanlab.cloud/DrugShot/117f31c00cef90ee68cebe3a3157a7748f82fad3/
Non-small cell lung cancer	0.84403	SIDER indications	https://appyters.maayanlab.cloud/DrugShot/60c28aff5fc5a731dae5aae5c77d17dec7d9e1a4/
Cervix carcinoma	0.844006	SIDER indications	https://appyters.maayanlab.cloud/DrugShot/2a58a9b18f1a0f67adab59d46129a8b81ad8bf3f/
Gastrointestinal carcinoma	0.8417	SIDER side effects	https://appyters.maayanlab.cloud/DrugShot/f96cce428065905c66c0ec59444316dcae44d717/
Impaired healing	0.8377	SIDER side effects	https://appyters.maayanlab.cloud/DrugShot/d6f0c132ceb3cb31301a64f2f8252554f3f2dd48/
Myeloid leukaemia	0.8361	SIDER side effects	https://appyters.maayanlab.cloud/DrugShot/c56dc625efee9bf409bf01429981bf0ee500ca4b/
Gastrointestinal toxicity	0.8348	SIDER side effects	https://appyters.maayanlab.cloud/DrugShot/9ec18f5d1415f37783c406a31c89740fc268f177/

### Atherosclerosis case study

Atherosclerosis is a common disease where cholesterol plaques accumulate in the inner walls of major arteries, ultimately leading to an artery blockage that can result in a stroke or a heart attack. There are several cholesterol-lowering drugs to treat the condition, including statins, niacin, PCSK9 inhibitors, fibrates, and adenosine triphosphate-citrate lyase (ACL) inhibitors. In this case study, we aim to demonstrate how DrugShot can be used to discover potential novel cholesterol-lowering drugs. We queried DrugShot with the terms “atherosclerosis”, “cholesterol”, and “statin” to find the drugs that are most associated with these terms in the literature, and then used the returned results to predict potential preclinical small molecules based on L1000 gene expression signature similarity (Additional file 1: Tables S1–S6). This approach assumes that preclinical small molecules that induce similar gene expression signatures in cell lines as the drugs currently used to lower cholesterol are likely to also have similar mechanisms of action (MOAs). In all searches, namely “atherosclerosis”, “cholesterol”, and “statin”, several preclinical LINCS-profiled drugs were ranked repeatedly in the top 20. These include: BMS-536924, SA-1478088, CAM-9-027-3, VU-0418939-2, purvalanol-a, methyl benzethonium, and TG-101348. Evidence exists that several of these preclinical compounds may have cholesterol-lowering activity. BMS-536924 is a competitive and selective insulin-like growth factor receptor (IGF-1R) kinase and insulin receptor (IR) inhibitor. Insulin-like growth factor (IGF) is involved in the proliferation and hypertrophy of vascular smooth muscle cells [22]. Previous studies reported that reduction in IGF-1 expression may be beneficial during early stages of plaque formation in atherosclerosis which is characterized by hypertrophy of vascular smooth muscle cells [23]. The third-ranked small molecule, SA-1478088, is a metalloproteinase inhibitor. Metalloproteinases are present in the extracellular environment of cells and are responsible for degradation of extracellular and intracellular proteins. Metalloproteinases have long been associated with atherosclerosis [24, 25], and metalloproteinase inhibitors such as SA-1478088 have been previously suggested as therapeutics for treating cardiovascular disease [26], which may suggest that SA-1478088 is a viable candidate for treating atherosclerosis. Hence, the highly ranked predicted compounds should serve as excellent candidates for *in-vitro* and *in-vivo* experiments of atherosclerosis disease models. This approach can be broadly applied to many other contexts.

## Discussion and conclusions

Here we present DrugShot, a web-server application and an Appyter to enable the ranking of drugs and small molecules based on any biomedical search term. DrugShot also makes predictions about additional small molecules that may be associated with the biomedical search term based on literature co-mentions or drug-drug LINCS L1000 gene expression signature similarity. The collection of compounds covered by DrugShot is mainly based on the ~ 20,000 LINCS L1000 compounds because these compounds have expression data needed for making predictions by DrugShot. Importantly, within these ~ 20,000 LINCS compounds, almost all FDA-approved drugs (small molecules) are included. However, it is possible to expand DrugShot’s chemical space. For example, we can use knowledge about the drugs' chemical similarity, known targets, results from bioassays, or other features, in addition to expression and literature co-mentions.

DrugShot also introduces DrugRIF and AutoRIF, two resources of drug-PMID associations. The drug-PMID co-occurrence identification by AutoRIF and DrugRIF is done with simple co-occurrence text processing (AutoRIF) or manual extraction of co-mentions (DrugRIF), which could benefit from more advanced text mining technologies that understand semantics and resolve synonyms, for example, BioBERT [27]. Furthermore, sets of small molecules and drugs can be submitted to DrugShot for drug set augmentation using the drug-drug similarity matrices. In this way, users can prioritize other compounds that may be related to their results from drug screens, or from annotated drug sets with a common effect or feature, for example, a shared side effect. Compared with the literature co-mentions matrices, the gene expression signature similarity matrix does not perform as well in predicting known compound-term associations. However, this does not mean that the predictions made by the LINCS L1000 gene expression signature similarity are less valid. A multitude of the small molecules in the gene expression signature similarity prediction matrix are experimental preclinical compounds, and their activity has not been fully characterized. Therefore, DrugShot may offer a method to prioritize these uncharacterized small molecules for experimental validation for specific applications. However, the predictions made by DrugShot are currently done by simple correlation calculations, and this can be improved in many ways. For example, such predictions can be replaced by more sophisticated machine learning methods that use other data modalities, non-linear kernels, and graph-based models. For example, Drugmonizome-ML [14] introduces a general framework for making such predictions systematically. Furthermore, by combining predictions with *in-vitro* drug screens, DrugShot predictions can be further calibrated. In the future, to maintain DrugShot, it is critical that DrugRIF and AutoRIF are continually updated, and the drug-drug similarity matrix based on gene expression is expanded. In addition, adding a feature that allows filtering chemicals by approval status may more readily highlight repurposing opportunities. It would also be interesting to see if DrugShot can predict marketed drug repurposing and marketed drug withdrawals before they occur. Importantly, in Table 3 we provide the most promising predictions to follow experimentally, as well as a short list of compounds that may be beneficial for atherosclerosis. These are just few examples to demonstrate the potential of DrugShot. Overall, expanding DrugRIF and AutoRIF with a larger compound pool, incorporating more drug-induced gene expression profiles into the signature similarity prediction matrix, adding additional drug-drug similarity matrices based on other attributes such as chemical similarity, and improving the methods used to make predictions by implementing more sophisticated methods, are different ways DrugShot can better illuminate associations between drugs and diseases, genes, and other biomedical and biological terms of interest.

### Availability and requirements

Project name: DrugShot

Project home page: https://maayanlab.cloud/drugshot/ and https://appyters.maayanlab.cloud/DrugShot/

Operating system(s): Platform independent

Programming language: Python

Other requirements: An up-to-date web browser

License: Apache License 2.0

## Supplementary Information

**Additional file 1.** **Supplementary Tables S1-S3.** **Supplementary Table S1**, small molecules associated with the search term "Atherosclerosis". **Supplementary Table S2**, small molecules associated with the search term "Statin". **Supplementary Table S3**, small molecules associated with the search term "Cholesterol". 

## Data Availability

The DrugShot web application is available at: https://maayanlab.cloud/drugshot. The DrugShot Appyter is available at: https://appyters.maayanlab.cloud/DrugShot/. DrugRIF is available at: https://appyters.maayanlab.cloud/storage/DrugShot/DrugRIF.tsv.gz. AutoRIF is available at: https://appyters.maayanlab.cloud/storage/DrugShot/AutoRIF.tsv.gz. DrugShot source code is available at: https://github.com/MaayanLab/drugshot and https://github.com/MaayanLab/appyter-catalog/tree/master/appyters/DrugShot.

## Abbreviations

LINCS: Library of Integrated Network-based Cellular Signatures
PMID: PubMed identifier
IUPAC: International Union of Pure and Applied Chemistry
InChIKey: IUPAC International Chemical Identifier Key
MeSH: Medical Subject Heading
NCBI: National Center for Biotechnology Information
ROC: Receiver operating characteristic
AUROC: Area under the receiver operating characteristic
ACL: Adenosine triphosphate-citrate lyase
MoA: Mechanism of action
IGF: Insulin-like growth factor
IR: Insulin receptor
