# Supplementary material for: DrugShot: querying biomedical search terms to retrieve prioritized lists of small molecules
Source: BMC Bioinformatics. 2022 Feb 19;23:76. doi: 10.1186/s12859-022-04590-5 (PMC8858480; doi:10.1186/s12859-022-04590-5)
Supplement: Supplementary file 1 — Additional file 1. Supplementary Tables S1-S3. Supplementary Table S1, small molecules associated with the search term "Atherosclerosis". Supplementary Table S2, small molecules associated with the search term "Statin". Supplementary Table S3, small molecules associated with the search term "Cholesterol". [file 12859_2022_4590_MOESM1_ESM.pdf]

S1. Tables of associated small molecules and predicted small molecules based on L1000 signature similarity from querying the term “Atherosclerosis”

Persistent Appyter Instance URL:
https://appyters.maayanlab.cloud/DrugShot/aa266ea234c983fdc385c9a57691848d9423d42f/

Rank	Drug Name	Publications with Search Term(s)	Publications w Search Term(s) / Total Publications
1	lovastatin	1131	0.0986
2	vitamin e	1125	0.0335
3	tidiacic arginine	1096	0.0186
4	aspirin	1094	0.0236
5	atorvastatin	1032	0.1497
6	angiotensin ii	958	0.0252
7	simvastatin	856	0.1067
8	ethanolamine	789	0.0037
9	estradiol valerate	635	0.0075
10	androstenol	556	0.0068
11	ferrous ascorbate	507	0.0116
12	pravastatin	493	0.1417
13	epoprostenol	463	0.0363
14	ezetimibe	453	0.1973
15	niacin	448	0.0412
16	folic acid	429	0.0109
17	ticlopidine	417	0.0369
18	rosuvastatin	381	0.1430
19	clopidogrel	371	0.0393
20	icosapent	365	0.0554

Table of top 20 associated small molecules produced from a DrugShot query with the term “Atherosclerosis”.

Rank	Drug Name	Score
1	CAM-9-027-3	0.1654
2	SA-1478088	0.1622
3	VU-0418939-2	0.1618
4	BMS-536924	0.1614
5	methyl-benzethonium	0.1585
6	BRD-K91663486	0.15574
7	IKK-16	0.15572
8	BRD-K73008154	0.15412
9	TG-101348	0.154
10	BRD-K63423329	0.1518
11	PP-110	0.1515
12	BRD-K30351863	0.1513
13	BRD-K87125912	0.1507
14	RS-17053	0.1497
15	BJM-CSC-15	0.14937
16	thapsigargin	0.14936
17	BRD-K41335306	0.149
18	BRD-K02025760	0.1478
19	BRD-K85211514	0.1469
20	BRD-K74461819	0.1465

Table of top 20 predicted small molecules associated with “Atherosclerosis” based on similarity of drug-induced gene expression signatures.

S2. Tables of associated small molecules and predicted small molecules based on L1000 signature similarity from querying the term "Statin"

Persistent Appyter Instance URL:
https://appyters.maayanlab.cloud/DrugShot/65beec341cde99f6fddf087108bcd492f11424a9/

Rank	Drug Name	Publications with Search Term(s)	Publications w Search Term(s) / Total Publications
1	lovastatin	11455	0.999
2	simvastatin	8009	0.998
3	atorvastatin	6894	1
4	pravastatin	3478	1
5	rosuvastatin	2665	1
6	ezetimibe	1609	0.7008
7	aspirin	1137	0.0245
8	fluvastatin	1056	0.7431
9	mevalonic acid	1052	0.2863
10	niacin	739	0.0679
11	pitavastatin	677	1
12	fenofibrate	533	0.1926
13	cerivastatin	526	1
14	mevastatin	457	1
15	ubidecarenone	395	0.0402
16	gemfibrozil	366	0.2633
17	ticlopidine	336	0.0297
18	clopidogrel	330	0.0350
19	clofibric-acid	328	0.0676
20	metformin	307	0.0204

Table of top 20 associated small molecules produced from a DrugShot query with the term “Statin”.

Rank	Drug Name	Score
1	BMS-536924	0.1808
2	PP-110	0.1757
3	CAM-9-027-3	0.175
4	TG-101348	0.1719
5	BRD-K91663486	0.1699
6	BRD-K41335306	0.1688
7	BRD-K63423329	0.1682
8	BRD-K87125912	0.1678
9	BRD-K73008154	0.1655
10	BRD-K74461819	0.1651
11	JW-7-24-1	0.165
12	BIBX-1382	0.1632
13	SA-1478088	0.1623
14	KU-0060648	0.1616
15	BRD-K49477330	0.1606
16	methyl-benzethonium	0.1601
17	BRD-K30351863	0.1585
18	IKK-16	0.1584
19	BRD-K85211514	0.1578
20	WYE-125132	0.1577

Table of top 20 predicted small molecules associated with “Statin” based on similarity of drug-induced gene expression signatures.

S3. Tables of associated small molecules and predicted small molecules based on L1000 signature similarity from querying the term "Cholesterol"

Persistent Appyter Instance URL:
https://appyters.maayanlab.cloud/DrugShot/1518fa951ce6f72bb00162fa7213cf0cdd07dd15/

Rank	Drug Name	Publications with Search Term(s)	Publications w Search Term(s) / Total Publications
1	lovastatin	5416	0.4723
2	simvastatin	3685	0.4594
3	androstenol	3450	0.0419
4	atorvastatin	3106	0.4505
5	vitamin e	3020	0.0899
6	estradiol valerate	2715	0.0320
7	phosphatidylethanolamine	1923	0.1482
8	pravastatin	1865	0.5362
9	ethanolamine	1840	0.0087
10	deoxycholic acid	1761	0.1477
11	ezetimibe	1736	0.7561
12	levothyroxine	1688	0.0343
13	mevalonic acid	1686	0.4588
14	ferrous ascorbate	1545	0.0355
15	ceramide	1458	0.1033
16	clofibric-acid	1440	0.2968
17	hydrocortisone	1364	0.0182
18	retinol	1270	0.0280
19	niacin	1226	0.1127
20	rosuvastatin	1154	0.4330

Table of top 20 associated small molecules produced from a DrugShot query with the term “Cholesterol”.

Rank	Drug Name	Score
1	VU-0418939-2	0.1567
2	SA-1478088	0.1489
3	CAM-9-027-3	0.14884
4	BMS-536924	0.14876
5	BG-1024	0.1464
6	purvalanol-a	0.1431
7	methyl-benzethonium	0.1401
8	BRD-K93623501	0.1389
9	IKK-16	0.1384
10	BRD-K87125912	0.13835
11	BRD-K85211514	0.13830
12	MG-132	0.1355
13	avicin-d	0.1351
14	5-nonyloxytryptamine	0.1346
15	BRD-K63423329	0.1341
16	thapsigargin	0.1334
17	chlorhexidine	0.1326
18	avermectin	0.1324
19	BRD-K14749055	0.13198
20	KU-0060648	0.13190

Table of top 20 predicted small molecules associated with “Cholesterol” based on similarity of drug-induced gene expression signatures.
